# Depressive symptoms, burnout, and declining medical career interest among undergraduate pre-medical students

**DOI:** 10.5116/ijme.5be5.8131

**Published:** 2018-11-26

**Authors:** Matthew K. Grace

**Affiliations:** 1 Department of Sociology, Hamilton College, Clinton, NY, USA

**Keywords:** Premedical education, mental health, medical career aspirations, depressive symptoms, burnout

## Abstract

**Objectives:**

To investigate the relationship between mental health issues and medical career interest among undergraduate premedical students, and to explore whether this association varies by gender.

**Methods:**

A longitudinal survey of freshman and sophomore premedical students at Indiana University was conducted during the 2015-16 academic year. Survey data were collected from 390 respondents via an online questionnaire (response rate=14%) in September 2015, and 292 of these respondents participated in the follow-up survey in April 2016 (retention rate=75%). Multi-level regression models were used to estimate the associations among depressive symptoms, burnout, and medical career interest.

**Results:**

Respondents who experienced more depressive symptoms (ß = -.07, z =-2.49, p =.013) and higher levels of burnout (ß = -.50, z =-3.98, p <.001) reported significant reductions in medical career interest over the study period. These associations remained consistent after controlling for socio-demographic characteristics and prior academic achievement. Depressive symptoms were associated with steeper declines in medical career interest among women compared to men (ß = -.09, z =-2.01, p =.045), though the relationship between burnout and medical career interest did not significantly vary by gender.

**Conclusions:**

Results provide evidence that premeds who experience more depressive symptoms and higher levels of burnout become less interested in entering the medical profession. The negative association between depressive symptoms and medical career interest was even more pronounced among premedical women compared to men. Findings suggest that colleges and universities in the United States should implement programs aimed at safeguarding the mental health of premedical students.

## Introduction

It is estimated that the United States will face a shortfall of nearly 120,000 physicians by 2030.^1^ In light of these projected shortages, there is a pressing need to identify upstream factors that contribute to the loss of otherwise qualified students from the medical education pipeline. To date, studies of pipeline issues in premedical education have primarily focused on illuminating the mechanisms responsible for the disproportionate attrition of students belonging to racial and ethnic minority groups.^2^^-^^4^ Other research on premedical education suggests that students are deterred from continuing their studies by a variety of considerations including the length of graduate medical training, trepidations about physicians’ long work hours, and the receipt of low grades in requisite coursework.^5^ Despite the notable contributions of these studies, much remains unknown about undergraduate premedical students and the factors that discourage their medical career aspirations.

A substantial body of empirical evidence indicates that graduate medical students experience high rates of depression, burnout, and suicidal ideation.^6^^-^^11^ While these statistics are troubling in their own right, it is also the case that medical students who experience mental health problems are significantly more likely than their peers to seriously contemplate a departure from medical school.^7^ Consistent with these findings, research on the mental health of premedical students finds that premeds experience more depressive symptoms and higher levels of burnout than their non-premedical counterparts.^12^^,^^13^ Yet, to date, it is unclear whether the mental health issues experienced by members of this group hold similar, negative consequences for their academic persistence. The answer to this question holds the potential to yield further insight into the psycho-social factors that predict leaks from the medical education pipeline at earlier points in the academic career.^14^

If mental health issues are negatively associated with medical career interest among premedical students, this association might not affect all premeds equally. Women in the United States experience internalizing forms of mental disorder (e.g., depression, generalized anxiety) more frequently than men.^15^ Corresponding with these broad social epidemiological patterns, past research indicates that premedical women are more susceptible than their male peers to both depression and burnout.^12^^,^^13^ It follows that if depressive symptoms and burnout are associated with diminished medical career aspirations, premedical women may be especially at risk of losing interest in entering the medical profession. Indeed, this relationship may help shed further light on why women are less likely than men to apply to medical schools in the United States despite being substantially more likely than men to arrive on college campuses aspiring to careers in medicine.^16^

The aims of this study were: (1) to investigate whether depressive symptomology and burnout are associated with medical career interest among undergraduate premedical students, and (2) to examine whether the relationship between mental health and medical career interest varies by gender. In light of the preceding discussion, it was hypothesized that premedical students who experience higher levels of depressive symptomology and burnout would become less interested in pursuing a career in medicine. Additionally, it was hypothesized that the negative association between mental health issues and medical career interest would be exacerbated among premedical women, such that higher levels of depressive symptomology and burnout among this group would be associated with greater declines in medical career interest.

## Methods

### Study design

This study draws upon longitudinal survey data collected from freshman and sophomore premedical students at Indiana University—a large public state university in the Midwestern United States—during the 2015-2016 academic year. Participants were recruited through the prehealth advising department’s e-mail listserv. All undergraduates who indicate an interest in applying to medical school during the admissions process are automatically registered to the prehealth advising department’s premedical e-mail listserv. Initial study invitations were sent via e-mail to all freshman and sophomore premedical students registered to the listserv in September of 2015 (Time 1). The invitation e-mail included a description of the study and a hyperlink to participate in the online survey (designed using Qualtrics survey platform). Respondents were offered a modest honorarium (a $10.00 Amazon gift card) in exchange for their participation. To supplement recruitment, a convenience sampling approach was also employed, with additional premedical students recruited from five introductory sociology courses at Indiana University, and granted extra credit in these courses in return for their participation (<11% of the sample was recruited using this supplemental method). All respondents who participated at Time 1 were invited to participate in a follow-up survey in April 2016 (Time 2) that was administered three-and-a-half weeks prior to the conclusion of the academic year. To ensure that each participant contributed only one unique response per survey wave, respondents were required to provide their university e-mail address in order to participate in the study. All aspects of the study were approved by the Indiana University Institutional Review Board and each respondent signed an electronic informed consent form prior to their participation, which acknowledged the voluntary nature of the study and their right to withdraw at any time.

### Participants

A total of 390 of 2,853 freshman (238/1,683) and sophomore (152/1,170) premedical students at Indiana University participated in the online survey in September 2015 (Time 1) for a response rate of approximately 14%. Of these initial respondents, 292 students elected to participate in the follow-up electronic survey that was conducted in April 2016 (Time 2), producing a study retention rate of 75%.

### Instruments

#### Medical Career Interest

The study’s focal dependent variable, medical career interest, was assessed at Time 1 and Time 2 using a modified version of an item used in past research on premedical students by Barr and colleagues 3 which instructed, “Please choose a whole number between 0 and 10 from the interest scale which best describes your current interest in becoming a physician.” Responses to this item were anchored at, (0) not at all interested, and (10) very interested.

#### Depressive symptoms

Depressive symptomology, one of the study’s key independent variables, was measured at Time 1 and Time 2 using the seven-item short form of the Center for Epidemiologic Studies of Depression Scale (CES-D).^17^^, ^^18^ This battery of questions asked respondents how frequently they experienced each of the following during the past month: “I did not feel like eating; my appetite was poor;” “I had trouble keeping my mind on what I was doing;” “I felt depressed;” “I felt that everything I did was an effort;” “My sleep was restless;” “I felt sad;” and, “I could not get ‘going’.” Responses for these items included (0) rarely or none of the time, (1) some or a little of the time, (2) occasionally or a moderate amount of time, and (3) most or all the time. A summative scale, ranging from 0 to 21, was created from responses to these items, with higher scores on the CES-D indicative of greater depressive symptomology (Alpha =.82 at Time 1, Alpha = .84 at Time 2).

#### Burnout

The study’s second key independent variable, burnout, was measured at both Time 1 and Time 2 using a fifteen-item version of the Maslach Burnout Inventory-Student Survey (MBI-SS) that was modified to reflect the study population.^19^^, ^^20^ The MBI-SS measures three dimensions of burnout among college students: exhaustion, cynicism, and low sense of professional efficacy. Items assessing exhaustion asked respondents to evaluate how frequently they felt the following: “I feel emotionally drained by my studies,” “I feel used up at the end of a day on campus,” “I feel tired when I get up in the morning and I have to face another day on campus,” “Studying or attending class is really a strain for me,” and “I feel burned out from my studies.” Items gauging cynicism asked respondents to indicate how often they felt the following: “I have become less interested in my studies since my enrollment at Indiana University,” “I have become less enthusiastic about my studies,” “I have become more cynical about the potential usefulness of my studies,” and “I doubt the significance of my studies.” Finally, low sense of professional efficacy was measured by reverse-coding responses to the following statements: “I can effectively solve the problems that arise in my studies,” “I believe that I make an effective contribution to the classes that I attend,” “In my opinion, I am a good student,” “I feel stimulated when I achieve my academic goals,” “I have learned many interesting things during the course of my studies,” and “During class I feel confident that I am effective in getting things done.” Response categories to all items included, (1) never, (2) a few times a year, (3) monthly, (4) a few times a month, (5) every week, (6) a few times a week, (7) everyday. A mean scale measuring burnout was created by averaging responses to items across the MBI-SS, with higher scores on the scale indicating greater experience of burnout (Alpha = .88 at Time 1, Alpha = .88 at Time 2).

#### Socio-demographic questionnaire

In addition to focal dependent and independent variables, data were also collected on participants’ sex, class year, race/ethnicity, prior academic achievement, family residence, and parental educational attainment. This information was used to create a series of covariates that were included as controls in regression models. Gender was reported as female or male. Class year was reported as freshman or sophomore. Respondents’ race/ethnicity (self-identified) was categorized as white, Asian American, black, Hispanic, and American Indian/Alaska Native/non-Hispanic multiracial. Past academic achievement was assessed by a self-reported measure of respondents’ high school cumulative grade point average (on a four-point scale). In line with a strategy employed by Barr and colleagues,^3^ the zip code of a respondent’s family residence was matched with data from the 2010 U.S. Census to create a measure of median household income. A log-transformed version of this variable was used for regression analyses. Finally, information on parental education was used to create a dichotomous variable measuring whether respondents were first-generation college students.

### Data analysis

Data analysis was performed using Stata 14.^21^ Descriptive statistics (means and standard deviations) were computed to summarize the characteristics of the sample, and two-sample Wilcoxon rank-sum tests were used to test for gender differences in depressive symptoms and burnout at Time 1 and Time 2. In order to evaluate the association between mental health and medical career interest and the possibility that this relationship varies by gender, a series of mixed-effects generalized linear regression models were estimated. Mixed-effects regression models are multi-level models for panel data that incorporate both random- and fixed-effects. In these models, observations (Level 1) from Time 1 and Time 2 are nested within individuals (Level 2) over time.^22^ A strength of this framework is that cases with missing data on a time-varying measure at one-time point can be retained in regression analyses without sacrificing consistency.^22^^,^^23^

Two other statistical adjustments warrant discussion. First, because women were overrepresented in the sample relative to the 2010 census of freshman premeds at Indiana University (69% versus 54%), post-stratification weights were implemented so that the gender composition of the analytic sample more closely resembled that of a standard cohort. Second, inverse probability weighting was used to adjust for bias stemming from the loss of 25% (n = 98) of the sample to follow-up.^24^ Inverse probability weights were created in a two-step process. In the first step, a logistic regression model predicting participation at Time 2 was estimated using the full Time 1 sample. Results from this model (full results available upon request) indicated that gender, class year, race/ethnicity, and working a job for pay did not have a significant association with respondents’ likelihood of participating in the follow-up survey. By contrast, respondents who attended private high schools (OR = .40, 95% CI = .19-.86, p = .018) and those who were recruited from sociology classrooms (OR = .21, 95% CI = .11-.40, p < .001) were significantly less likely to participate at Time 2. To adjust for these sources of differential attrition, predicted probabilities were generated from this model and used to create analytic weights in inverse proportion to the probability of a respondents’ likelihood of participating at Time 2. This process assigned greater weight to the data provided by respondents who were statistically the least likely to participate at Time 2. Although this strategy cannot fully adjust for the bias introduced by differential attrition, it provides a closer approximation of what regression results would look like had these respondents been retained. For the longitudinal analyses presented here, cases were restricted to respondents who participated in both survey waves (n=292). Several additional cases were removed because these respondents failed to provide information that was used to create control variables (n=6), leaving a total analytic sample of 286 respondents.

## Results

### Socio-demographic characteristics of premedical students

Descriptive statistics for the analytic sample are presented in Table 1. Of the 286 respondents in the sample, 196 (69 %) were female, and 90 (31%) were men. 165 (58%) were freshmen compared to 121 (42%) sophomores. In terms of racial/ethnic composition, most respondents were non-Hispanic white (199 respondents, 70%), 23 (8%) were Black, 34 (12%) were Asian American, 22 (7%) were Hispanic, and 8 (3%) were American Indian, Alaska Native, or multiracial (non-Hispanic). The most recent unofficial census of freshman premedical students conducted at Indiana University in 2010 found that this cohort was roughly 61% white, 6% black, 11% Asian American, and 5% Hispanic (17 % not ascertained). Thus, the sample roughly approximates the racial/ethnic composition of a typical premedical cohort at Indiana University. Turning to other respondent characteristics, participants had relatively strong high school grades (M = 3.78 on a four-point scale) and came from communities where the median household income is approximately $66,735 (SD = $25,442). The sample included 55 (19%) first-generation college students.

**Table 1:** Descriptive statistics for key study variables and respondent characteristics of the analytic sample (N=286)

Key study variables	Mean %	SD
Dependent variable		
Medical career interest, T1	8.42	1.92
Medical career interest, T2	7.61	2.94
Independent variables		
Depressive symptoms, T1	6.88	4.34
Depressive symptoms, T2	8.45	4.71
Burnout, T1	2.83	.93
Burnout, T2	3.28	.98
Respondent characteristics		
Gender		
Male	31	
Female	69	
Class year		
Sophomore	42	
Freshman	58	
Race/Ethnicity		
Non-Hispanic white	70	
Black	8	
Asian American	12	
Hispanic	7	
American Indian/Alaska Native/non-Hispanic multiracial	3	
Academic achievement		
High school grade point average	3.78	.30
Socioeconomic status		
Median neighborhood income	66,735	25,442
First generation college student	19	

Note: Unweighted means with standard deviations in parentheses. T1=September 2015, T2=April 2016.

### The relationship between mental health and medical career interest

Table 2 presents results from a series of mixed-effects regression models that examine the associations of depressive symptoms and burnout with medical career interest. Model 1 shows the bivariate association between depressive symptoms and medical career interest net of adjustments for non-random attrition and post-stratification weights for gender. Results from this model indicated that higher levels of depressive symptomology were associated with significant declines in medical career interest (ß = -.07, z = -2.49, p = .013). When controls for respondent characteristics were added in model 2, the relationship between depressive symptoms and medical career interest remained substantively the same (ß = -.06, z = -2.34, p = .019). Turning to the relationship between burnout and medical career interest, a similar pattern unfolds. In model 3, which adjusted for differential attrition and included post-stratification weights, higher levels of burnout (ß = -.50, z = -3.98, p < .001) were negatively associated with medical career interest. When controls for socio-demographic characteristics and past academic achievement were introduced into the regression equation in model 4, the negative relationship between burnout and medical career interest remained consistent (ß = -.50, z = -3.95, p < .001).

### Gender differences in the relationship between mental health and medical career interest

Results from two-sample Wilcoxon rank-sum tests showed that women reported more depressive symptoms and higher levels of burnout than men at both Time 1 (respectively, z = -2.61, p = .0091; and z = -2.98, p = .0029) and Time 2 (respectively, z = -2.39, p .017; and z = -2.31, p = .021). To test whether the associations of depressive symptoms and burnout with medical career interest significantly varied by gender, the dichotomous variable for gender was interacted with depressive symptoms and burnout in separate mixed-effects regression models predicting medical career interest. As shown in model 1 of Table 3, there was a significant, negative interaction between gender and depressive symptoms (ß = -.09, z = -2.01, p = .045). This coefficient indicates that women who reported more depressive symptoms over the course of the academic year experienced significantly greater declines in medical career interest compared to their male peers. A significant interaction was not found between gender and burnout in model 2.

## Discission

Efforts to understand the antecedents of premedical students’ declining interest in medicine are increasingly relevant in light of projected physician shortages in the United States. Following from research documenting high levels of depression^12^and burnout^13^among premedical students—factors that have been associated with thoughts of medical school departure among graduate medical students^7^—this study sought to examine whether these facets of mental health were associated with chang-es in medical career aspirations among premeds. Fur-thermore, because depressive symptoms and burnout are more common among premedical women compared to men,^12^^,^^13^ a secondary aim of this study was to examine whether the association between mental health and medical career interest significantly varied by gender.

**Table 2:** Mixed-effects regression models estimating the associations of depressive symptoms and burnout with medical career interest (N=286)

Variables	Model 1	Model 2	Model 3	Model 4
Depressive Symptoms	-.07*	-.06*		
	[.03]	[.03]		
Burnout			-.50***	-.50***
			[.13]	[.13]
Gender^1^				
Female		-.60*		-.50*
		[.25]		[.24]
Class Year ^2^				
Freshman		-.47		-.57*
		[.26]		[.25]
Race/Ethnicity^3^				
Black		1.26**		1.14**
		[.43]		[.42]
Asian-American		.03		.14
		[.41]		[.40]
Hispanic		.06		-.01
		[.52]		[.51]
American Indian / Alaska		.63		.46
Native / Non- Hispanic Multiracial		[.69]		[.71]
Academic Achievement				
High school grade point		1.09*		1.00*
Average		[.47]		[.48]
Socioeconomic status				
Median income (log)		.16		.08
		[.32]		[.31]
First generation college		.01		-.14
Student^4^		[.34]		[.32]
Time^5^	-.65***	-.65***	-.54***	-.54***
	[.15]	[.15]	[.15]	[.14]
BIC	2550.11	2588.92	2528.91	2566.81

Notes: Unstandardized beta coefficients presented with standard errors in brackets. All models include post-stratification weights for gender and inverse probability weights to adjust for non-response at Time 2. Model constant and random-effects not shown. 1 Male is referent group; 2 Sophomore is referent group; 3 White is referent category for race/ethnicity; 4 Non-first-generation student is referent group; 5 Time is coded as 0=September 2015, 1=April 2016.

*p<.05; **p<.01; ***p<.001 (two-sided tests).

Using longitudinal survey data collected from early career premedical students at Indiana University, results showed that respondents who experienced more depressive symptoms and higher levels of burnout over the course of the academic year reported significant reductions in medical career interest. This finding broadly corresponds with research on graduate medical education, which indicates that medical students who experience greater burnout and depressive symptomology are more likely to seriously contemplate dropping out of medical school.^7^ The premedical track in the United States is an academically demanding pre-professional path characterized by intensive coursework in chemistry, biology, and physics, and competition with peers for one of a limited number of medical school seats in a given admissions cycle.^5^^,^^14^^,^^25^Successful applicants to medical school frequently combine high grades in difficult coursework with a carefully curated list of time-consuming extracurricular activities.^26^^,^^27^For some pre-meds, this combination of academic and social stressors may combine to erode their psychological well-being and weaken their resilience in the face of premedical track-related obstacles—a dynamic that could help to explain the relationship between higher levels of depressive symptoms/burnout and greater declines in medical career interest observed in this study.

**Table 3:** Coefficients from mixed-effects regressions of medical career interest on models with interactions of depressive symptoms/burnout x gender (N=286)

Variable	Model 1	Model 2
Female x depressive symptoms	-.09*	
	[.05]	
Female x Burnout		-.02
		[.24]
Depressive symptoms	.00	
	[.04]	
Burnout		-.49*
		[.20]
Gender^1^		
Female	.08	-.44
	[.40]	[.68]
Class year^2^		
Freshman	-.44	-.57*
	[.27]	[.25]
Race/Ethnicity^3^		
Black	1.29**	1.14**
	[.42]	[.43]
Asian-American	.02	.13
	[.41]	[.40]
Hispanic	.04	-.01
	[.51]	[.51]
American Indian / Alaska	.68	.46
Native / Non- Hispanic multiracial	[.71]	[.71]
Academic achievement		
High school grade point	1.12*	1.00*
Average	[.46]	[.48]
Socioeconomic status		
Median income (log)	.17	.08
	[.32]	[.31]
First generation college	.05	-.14
Student^4^	[.34]	[.33]
Time^5^	-.66***	-.55***
	[.15]	[.15]
BIC	2590.93	2573.15

Notes: Unstandardized beta coefficients presented with standard errors in brackets. All models include post-stratification weights for gender and inverse probability weights to adjust for non-response at Time 2. Model constant and random-effects not shown. Male is referent group; 2 Sophomore is referent group; 3 White is referent category for race/ethnicity; 4 Non-first-generation student is referent group; 5 Time is coded as 0=September 2015, 1=April 2016.

*p <.05; **p <.01; ***p <.001 (two-sided tests).

A growing body of research has highlighted troubling rates of depression, burnout, and suicidal ideation among graduate medical students.^8^^-^^10^ In response, researchers and administrators have argued that measures must be taken to address the mental health of this group. The findings of the present study suggest that similar attention must also be paid to the mental health of undergraduate premedical students. While colleges and universities in the United States increasingly offer academic advising tailored to the specific needs of premedical students, findings indicate that these services should be expanded to include a focus on students’ mental health. On campuses where mental health services exist, premedical advisors and faculty should coordinate with these offices for instruction in how to identify symptoms of depression and burnout among students. These efforts could be further strengthened by establishing a formal protocol for directing students identified as being at-risk for depression or burnout to treatment. Given the high degree of the stigma surrounding mental health issues found among medical trainees,^10^ this endeavor will undoubtedly require intensive efforts by academic counselors to normalize the use of mental health services during their meetings with premedical students.

Aligning with past research on premedical students,^12^^,^^13^premedical women reported significantly higher levels of depressive symptoms and burnout than men at both survey waves in this study. Interaction models assessing whether the association between mental health issues and medical career interest varied by gender indicated that depressive symptoms were associated with greater declines in medical career interest among premedical women compared to men. Although women have only recently surpassed men in medical school admissions in the United States,^28^ there is evidence from nationally representative data on freshmen at four-year colleges and universities that women have been more likely than men to arrive on college campuses aspiring to careers in medicine since 1994.^16^The stronger negative association between depressive symptoms and medical career aspirations among women may provide further insight into why roughly similar numbers of men and women apply to American medical schools each year despite women being considerably more likely to report an intention of applying to medical school as college freshmen.^29^ Past research on women in science, technology, engineering, and mathematics finds that positive relationships with faculty, as well as close relationships with same-sex peers, can help to retain women in these fields.^30^Consequently, mentor programs that pair premedical women with supportive faculty, or initiatives aimed at cultivating relationships among women enrolled in the same coursework, may help to sustain women’s medical career ambitions in the face of burgeoning mental health issues.

Findings from this study should be interpreted in light of several limitations. Most apparent are those stemming from the study’s low response rate and focus on a single research site. In terms of the former, declining response rates are increasingly common in survey research.^31^^,^^32^ Although the honorarium offered to participants was increased to encourage participation; financial limitations ultimately placed a limit on further efforts to incentivize participation. Regarding the latter, the study’s focus on a large state university limits the generalizability of findings to other institutional settings. Indeed, while mental health issues may be associated with declining medical career interest among premeds at large public universities, it is plausible that this relationship may unfold differently at small liberal arts colleges or historically black universities where there are demonstrable differences in demographic composition, class size, and institutional missions. Response bias is another important issue to consider. Because demographic information for the students on the premedical listserv who chose not to participate in the study was not granted by Indiana University, it was not possible to discern whether there were substantive differences between premeds who elected to participate in the study and those who did not. Future studies should focus on research sites where demographic information on the premedical population is more thoroughly documented so statistical analyses can be weighted to account for respondent characteristics that predict selection into the sample.

## Conclusions

Results from this prospective case study provide preliminary evidence that undergraduate premedical students who experience more depressive symptoms and higher levels of burnout become less interested in pursuing a career in medicine. The relationship between depressive symptoms and declining medical career interest was even more pronounced among premedical women compared to men. Albeit suggestive due to its small sample size and focus on a single research site, results from this study indicate that failure to provide adequate mental health services to undergraduate premedical students may lead to the siphoning of otherwise qualified premedical students from the medical education pipeline. These findings point to the need for a multi-site, nationally representative study of premedical education that can more definitively speak to the influence of mental health problems on undergraduates’ medical career aspirations.

### Acknowledgements

This research was generously supported by the National Science Foundation through a Dissertation Improvement Grant No.1519056.

### Conflict of Interest

The authors declare that they have no conflict of interest.
